# Epigenome-wide analysis identifies DNA methylation signatures associated with the infant pupillary light reflex, a candidate intermediate phenotype for autism

**DOI:** 10.1038/s41598-025-31651-5

**Published:** 2026-01-08

**Authors:** Laurel A. Fish, Teodora Gliga, Anna Gui, Jannath Begum Ali, Luke Mason, Mark H. Johnson, Tony Charman, Terje Falck-Ytter, Emily J. H. Jones, Radhika Kandaswamy, Francesca Happé, Chloe C. Y. Wong

**Affiliations:** 1 Department of Clinical, Educational and Health Psychology, University College London, London, UK; 2 Social, Genetic and Developmental Psychiatry Centre, Institute of Psychiatry, Psychology & Neuroscience, King’s College London, London, UK; 3 School of Psychology, University of East Anglia, Norwich, UK; 4 University of Rome Tor Vergata, Rome, Italy; 5 Department of Psychological Sciences, Birkbeck College, University of London, London, UK; 6 Department of Psychology, Cambridge University, Cambridge, UK; 7 Department of Psychology, Institute of Psychiatry, Psychology & Neuroscience, King’s College London, London, UK; 8 Development and Neurodiversity Lab, Department of Psychology, Uppsala University, Uppsala, Sweden; 9 Department of Women’s and Children’s Health, Center of Neurodevelopmental Disorders at Karolinska Institutet (KIND), Karolinska Institutet, Stockholm, Sweden; 10 UK National Institute for Health and Care Research Biomedical Research Centre for Mental Health, South London and Maudsley Hospital, London, UK; 11 Department of Child & Adolescent Psychiatry, Institute of Psychiatry, Psychology & Neuroscience, King’s College London, London, United Kingdom

**Keywords:** Pupillary light reflex (PLR), Epigenetics, DNA methylation, Autism, Intermediate phenotype, Genetics, Neuroscience, Psychology, Biomarkers, Signs and symptoms

## Abstract

The pupillary light reflex (PLR), the automatic constriction of the pupil in response to increased luminance, is a candidate early intermediate phenotype associated with autism, with potential to help understand early neurodevelopmental differences because it is controlled by relatively simple neural circuitry. We conducted epigenome-wide association analyses of PLR onset latency and constriction amplitude at 9, 14, and 24 months, with 51 male infants enriched for familial autism likelihood (~ 80% with a first-degree autistic relative), using buccal DNA collected at 9 months. We identified four epigenome-wide differentially methylated probes (*p <* 2.4 × 10⁻⁷) significantly associated with PLR latency at 14 and 24 months, and 14- to 24-month developmental change in latency. Probes linked to PLR amplitude were identified at a discovery threshold (*p <* 5 × 10⁻⁵). Regional analyses revealed multiple differentially methylated regions associated with both latency and amplitude. Associated probes were enriched for neurodevelopmental processes and autism-associated genes, including *NR4A2*, *HNRNPU*, and *NAV2*. While the findings are most directly relevant to male infants in whom PLR variability may be associated with familial autism likelihood, they provide novel evidence that DNAm contributes to early variation in PLR. These insights into the biological underpinnings of this reflex support PLR as an early intermediate phenotype associated with autism.

**Supplementary Information:**

The online version contains supplementary material available at 10.1038/s41598-025-31651-5.

## Introduction

Autism is a life-long, highly heterogeneous neurodevelopmental condition characterised by differences in social interactions and communication, alongside specialised, focused, or intense interests, repetitive behaviours and sensory differences^1^. Autism cannot be diagnosed reliably until two years, at the earliest, when behavioural characteristics are sufficiently developed for clinical observation^2^. Prospective investigation of early intermediate phenotypes, defined as pre-diagnosis measurable features associated with the later behavioural characteristics^3^, may help clarify early neurodevelopmental pathways underlying autistic traits^4^ and ultimately inform research into supportive strategies for autistic people.

The pupillary light reflex (PLR) is a candidate intermediate phenotype associated with autism^5^. The PLR is the reflexual constriction of the pupil in response to increased optical luminance^6^ and is primarily mediated by a relatively simple four-neuron visual sensorimotor circuitry^7^. This well-studied index of autonomic functioning is governed by parasympathetic and sympathetic pathways^8^ and its regulation involves neural signal transduction and cholinergic and norepinephrine neurotransmission^8–12^. Recent findings indicate that variability in infant PLR associates with both categorical autism diagnosis and dimensional variation in later autism traits, making it a useful measure for studying early differences in neurodevelopment. Cross-sectionally, 10-month-old infants with a family history of autism have a larger and faster PLR relative to infants with no family history^13^, with larger 10-month PLR constriction amplitude associating with later autism diagnosis^14^. Developmentally, from 6 to 24 months, PLR tends to become faster and larger^15,16^, but a decreasing pattern of pupil constriction amplitude over time in those who later receive an autism diagnosis has been demonstrated^14^. This finding was replicated in a partially overlapping sample in which a decrease in amplitude between 9 and 14 months was associated with autistic traits^5^. In the same sample, those with a later diagnosis demonstrated a significantly steeper decrease in latency from 14 to 24 months relative to those with other outcomes^5^. Additionally, a higher polygenic score for autism associated with a smaller decrease in latency in the first year^5^.

Increasing evidence suggests autism arises during early development from complex interactions between genetic and environmental factors^17^. Though the path through which these factors coalesce remains unclear, emerging evidence points to altered epigenetic signatures in autism^18^. Epigenetic modifications, such as DNA methylation (DNAm), are chemical markers that can influence gene expression without altering the underlying DNA sequence itself^19^. DNAm is a key biological mechanism involved in the timed up- or down-regulation of gene expression^20,21^. Recent epigenome-wide association studies (EWAS) using both post-mortem brain tissue and peripheral tissue (e.g. blood, buccal or saliva) from living participants have identified several differentially methylated sites and regions, as well as co-methylated modules, associated with both autism diagnosis^22–24^ and dimensional autism traits^25^. Autism-associated DNAm signatures have been identified in genes and pathways linked to critical functional processes, including neurodevelopment, immune response, synaptic functioning, and microscopic neural structures; highlighting their potential and relevance for providing insight into early neurodevelopmental variation related to autism^22–24,26,27^.

Notably, most prior studies have used samples taken post-diagnosis. Since autism is a neurodevelopmental condition, prospectively investigating DNAm before diagnosis could be fruitful in mapping early autism development. A recent EWAS^28^ identified differential peripheral tissue DNAm in 8-month-olds associated with 3-year autism diagnosis and dimensional traits. However, these associations were not statistically significant after stringent epigenome-wide multiple testing corrections. An alternative, potentially more statistically powerful, method of exploring the role of DNAm in early autism is to investigate the association of DNAm with early intermediate phenotypes^4^, which tend to be placed at an earlier stage in the developmental pathway^3^. Indeed, an EWAS with two social attention candidate 8-month intermediate phenotypes for autism (face looking and neural response to faces) was recently conducted, though, again, no probe-wise associations were significant after epigenome-wide multiple testing corrections^28^. Given that the pupillary light reflex (PLR) is a quantifiable sensorimotor reflex involving relatively simple neuro-circuitry, it is likely situated earlier in the developmental pathway than other candidate intermediate phenotypes. This offers promise for exploring how epigenetic variation relates to early neurodevelopmental differences.

In this study, we aimed to expand previous research indicating PLR as a candidate intermediate phenotype for autism^5^ by exploring the epigenetic variability associated with individual differences in early PLR development. We present findings from epigenome-wide DNAm association analysis of infant PLR onset latency and constriction amplitude, using buccal tissue taken from 51 male 9-month-old infants enriched for autism family history. A minority of infants (20%) without a family history of autism were included to broaden PLR variability and enable examination of epigenetic differences across the full range of this candidate intermediate phenotype. We applied a hypothesis-free epigenome-wide approach to explore the association of probe- and region-level DNAm with variability in cross-sectional (9-, 14-, and 24-month) and developmental (9- to 14-month and 14- to 24-month changes) PLR onset latency and constriction amplitude. We conducted downstream gene ontology enrichment analysis to uncover biological significance and performed functional exploratory analyses to investigate genetic and epigenetic associations with autism.

## Results

### DNAm analysis

Our study identified multiple novel significantly differentially methylated probes and regions associated with PLR latency and amplitude. A summary of significant results from the epigenome-wide association studies (EWAS) and differentially methylated region (DMR) analyses is presented in Table 1 (SM 4.1 includes Q-Q plots and EWAS Manhattan plots).

**Table 1:** Summary of probe-wise EWAS and DMRs identified for each phenotype.

Phenotype	*N*. participants	λ	PLR-associated DMP analysis		PLR-associated DMR analysis
			*N*. stringent-sig. probes (*p* < 2.4 × 10^− 7^)	*N*. discovery-sig. probes *(p* < 5 × 10^− 5^)	*N*. Bonf.-adj. sig. DMR (*p* > 0.05)
Latency (ms)					
9 months	48	1.16	0	28	1
14 months	47	1.14	2	27	1
24 months	40	1.02	1	21	1
9 to 14 months	44	0.94	0	6	1
14 to 24 months	37	1.20	1	60	9
Amplitude (%)					
9 months	46	1.07	0	11	1
14 months	47	1.03	0	21	1
24 months	37	1.05	0	28	5
9 to 14 months	42	1.06	0	28	3
14 to 24 months	34	0.96	0	15	7

*λ* genomic inflation factor, *sig.* significant, *Bonf.-adj* Bonferroni-adjusted.

### PLR latency

We identified four highly significant differentially methylated probes associated with three PLR latency phenotypes at a stringent p-value threshold (*p* < 2.4 × 10^− 7^, see Table 2). A broader list of probes associated with PLR latency phenotypes above the discovery p-value threshold (*p* < 5 × 10^*− 5*^) is provided in SM 4 Table 1.

**Table 2:** Differentially methylated probes associated (*p* < 2.4 × ***10***^***− 7***^) with PLR latency.

Probe	Latency phenotype (months)	Genomic locations (hg19)	Illumina gene annotation	Effect size (β)^1^	*p*-value
cg05148717	14	chr6:28829171		−0.00055	3.74 × 10^− 8^
cg22367466	14	chr12:6840532	*COPS7A*	−0.00035	4.28 × 10^− 8^
cg09732535	24	chr16:89331997		0.00066	1.85 × 10^− 8^
cg15130433	14 to 24	chr6:159240081	*EZR*	0.00025	2.21 × 10^− 7^

^1^A negative effect size (-β) indicates hypermethylation associated with faster latency (for cross-sectional measurements) or latency becoming slower overtime (for change scores).

In total, we reported 13 DMRs significantly associated with PLR latency; each latency phonotype had a single significantly associated DMR, except for 14- to 24-month PLR latency change, where nine significantly associated DMRs were found. The DMRs significantly associated with each PLR latency phenotype are listed in Table 3 and SM 4 Table 2.

**Table 3:** Summary of the significantly associated DMRs for each PLR latency phenotype.

Latency phenotype (months)	Genomic locations (hg19)	*N* probes	Probes	Illumina gene annotations	Effect size (β)^1^	Bonf.-adjust *p*-value
9	chr10:74927623–74,927,863	8	cg15213114; cg04167018; cg21416602; cg25138168; cg08571229; cg04749667; cg16124546; cg12276298	*FAM149B1; ECD*	−0.007	7.25 × 10^− 3^
14	chr1:103573700–103,573,772	2	cg26436330; cg20847625	*COL11A1*	0.01	6.33 × 10^− 4^
24	chr:1045495981-45496216	3	cg18382353; cg16512882; cg15078013	*C10orf25; ZNF22*	−0.02	8.49 × 10^− 4^
9 to 14	chr3:52569053–52,569,169	3	cg18337363; cg08365687; cg13284614	*NT5DC2; LOC440957*	−0.009	7.35 × 10^− 3^
14 to 24^2^	chr17:2699706–2,699,718	2	cg05890550; cg25373595	*RAP1GAP2*	0.02	6.53 × 10^− 5^

*Bonf.-adjust* Bonferroni-adjusted. ^1^A negative effect size (-β) indicates hypermethylation associated with faster latency (for cross-sectional measurements) or latency becoming slower over time (for change scores).^2^This row details the topmost significant DMR to associate with 14- to 24-month latency change (see SM 4 Table 2 for a summary of all DMRs significantly associated with PLR latency).

### PLR amplitude

In contrast to PLR latency, PLR amplitude did not show probe-level associations at epigenome-wide significance after stringent correction for multiple comparisons (*p* < 2.4 × 10^− 7^). Several differentially methylated PLR amplitude-associated probes reached the discovery p-value threshold (*p* < 5 × 10^− 5^; See full list in SM 4 Table 1). Region analyses revealed 17 DMRs significantly associated with PLR amplitude, with the topmost significantly associated DMR per phenotype listed in Table 4 (full list in SM 4 Table 2).

**Table 4:** Summary of the topmost significantly associated DMRs for each PLR amplitude phenotype.

Amplitude phenotype (months)	Genomic locations (hg19)	*N* probes	Probes	Illumina gene annotation	Effect size (β)^1^	Bonf.-adjust *p*-value
9	chr13:103452556–103,453,215	4	cg06518779; cg21251000; cg15186648; cg15193473	*BIVM; KDELC1*	0.04	2.13 × 10^− 4^
14	chr11:111249659–111,250,201	5	cg19126910; cg17390301; cg24049888; cg18316498; cg11362935	*POU2AF1*	−0.03	2.22 × 10^− 2^
24	chr5:77253833–77,253,990	3	cg25051331; cg09048251; cg07595776		−0.08	8.23 × 10^− 4^
9 to 14	chr10:102295134–102,295,549	5	cg07690778; cg26303175; cg07080220; cg08314679; cg07510080	*HIF1AN*	0.07	2.67 × 10^− 3^
14 to 24	chr15:89786761–89,787,223	4	cg22813622; cg01741397; cg15769724; cg06870609	*FANCI*	−0.04	1.22 × 10^− 3^

*Bonf.-adjust* Bonferroni-adjusted. ^1^A negative effect size (-β) indicates hypermethylation associated with smaller constriction amplitude (for cross-sectional measurements) or constriction becoming larger over time (for change scores).

### Downstream exploratory analysis

Gene ontology (GO) analysis was conducted for each phenotype using genes annotated with probes within the top 1000 EWAS p-values, and probes located within the significantly associated DMRs. Multiple significantly enriched GO terms were identified (Table 5). SM 5 Table 2 lists all significantly enriched GO terms.

**Table 5:** Summary of the number of significantly enriched GO terms and the most significant term for each PLR phenotype.

Phenotype (month)	*N*. sig. enriched GO terms	Most significantly enriched GO term			
		Description (GO number)		Fold Enrichment	FDR *p*-value
PLR latency					
9	12	Homophilic cell adhesion via plasma membrane adhesion molecules (GO:0007156)		4.2	3.17 × 10^− 7^
14	34	Negative regulation of developmental process (GO:0051093)		1.84	6.95 × 10^− 3^
24	0	-		-	-
9 to 14	16	Homophilic cell adhesion via plasma membrane adhesion molecules (GO:0007156)		6.62	8.27 × 10^− 22^
14 to 24	16	Regulation of cellular component organization (GO:0051128)		1.5	5.00 × 10^− 3^
PLR amplitude					
9	0	-		-	-
14	73	Regulation of glucuronosyltransferase activity (GO:1904223)		24.41	3.86 × 10^− 8^
24	0	-		-	-
9 to 14	22	System development (GO:0048731)		1.41	1.30 × 10^− 3^
14 to 24	0	-		-	-

*sig.* significant after False discovery rate (FDR) adjustment.

For both PLR components, no probes above the discovery p-value threshold, nor probes located in any of the significantly associated DMRs, were listed in the MRC-IEU EWAS catalogue^29^. None of the stringently significant probes were annotated to genes listed in the SFARI database^30^. Nineteen probes significantly associated with PLR at the discovery p-value threshold were annotated to 18 distinct genes listed in the SFARI gene. Notably, two SFARI genes with ‘high confidence’ associations were annotated with probes identified to significantly associate with latency at 14 months (cg01123282; *NR4A2*) and latency at 24 months (cg02230180; *HNRNPU*). A full list of probes annotated to SFARI genes is summarised in SM 5 Table 3.

Three of the identified significantly associated DMRs contained probes annotated to three distinct SFARI genes, as summarised in Table 6.

**Table 6:** Summary of significantly associated DMRs annotated to genes previously associated with autism.

DMR genomic locations (hg19)	Probes	Associated phenotype	Gene	SFARI gene-score^1^
chr11:19372012–19,372,234	cg23330281; cg24137774; cg25909885	Latency 14 to 24 month	*NAV2*	2 (strong candidate)
chr7:2968559–2,968,595	cg22989995; cg23770265	Amplitude 24 month	*CARD11*	2 (strong candidate)
chr13:38444227–38,444,490	cg16409955; cg23021771	Amplitude 9 to 14 month	*TRPC4*	3 (suggestive evidence)

^1^SFARI gene-scores is a ranking system that reflects the overall strength of evidence of the gene’s relevance to autism^30^.

## Discussion

The infant pupillary light reflex (PLR) is increasingly recognised as a candidate early intermediate phenotype for autism, offering a potential window into the neurobiological pathways underlying autism. In this study, we aimed to expand previous work by exploring the epigenetic variability associated with the early development of the PLR in a sample of male infants enriched for familial autism likelihood. This sample included a small proportion (20%) of infants with no autism family history to introduce a modest degree of phenotypic heterogeneity. This was advantageous for capturing broader, but still relevant, variability in PLR to better assess associated DNA methylation (DNAm) variation in this candidate intermediate phenotype. Our hypothesis-free epigenome-wide scan identified four novel Bonferroni-significant (*p* < 2.4 × 10 − 7) differentially methylated probes and 13 differentially methylated regions (DMRs) associated with PLR latency phenotypes, particularly within a sensitive neurodevelopmental window between 14 and 24 months. These findings provide compelling evidence that differential epigenetic signatures contribute to the variability of early PLR onset latency.

In contrast, PLR amplitude did not show probe-level associations at epigenome-wide significance after Bonferroni corrections. This divergence between PLR components likely reflects their underlying biological differences. Specifically, while the PLR is driven by a core four-neuron reflex circuit^7^, increasing evidence suggests that PLR amplitude is modulated by additional top-down higher-order cognitive processes, including emotional regulation and executive control^31–34^. Supporting this, our downstream GO analysis indicated that probes associated with amplitude at discovery significance threshold (*p* < 5 × 10⁻⁵) were annotated to a broader and more complex biological network than latency-associated probes. While amplitude-related terms shared some overlap with those of latency, such as system development (GO:0048731) and homophilic cell adhesion via plasma membrane adhesion molecules (GO:0007156), amplitude-related terms also included categories related to cell signalling (e.g., cell-cell signalling, GO:0007267), metabolic processes (e.g., cellular glucuronidation, GO:0052695), and stress response (e.g., regulation of response to stimulus, GO:0048583). This more diffuse regulatory landscape may dilute the detectable effects of single-probe DNAm associations for amplitude. In contrast, if the PLR latency is controlled by a tightly focused, mostly bottom-up biological system, then DNAm changes in that system might have stronger and more specific effects. Because the effects would be concentrated in a small, well-defined pathway rather than spread across many brain systems, they’re more likely to be statistically significant even under the very strict Bonferroni correction used in our EWAS analysis.

Both PLR latency and DNAm have previously been linked to early autism characteristics, particularly sensory sensitivities^5,13,28^. To explore the connection of the identified epigenetic signatures of the PLR latency with autism, we undertook an in silico experiment to examine whether associated probes or regions were listed in the SFARI database^30^ or the EWAS catalogue^29^. While none of the Bonferonni-corrected probes or regions had prior links to autism, as reported in these two databases, several discovery-significant probes across the five latency EWASs were annotated to autism susceptibility genes. Among the most compelling genes were *NR4A2* and *HNRNPU*, listed as ‘high confidence’ autism-associated genes in the SFARI database given the multiple separate reports identifying mutations in each of these genes in autistic people^35–39^. Specifically, probe cg01123282, located in intron 1 of *NR4A2*, was hypermethylated in association with slower latency at 14 months. Probe cg02230180, located in intron 1 of HNRNPU, was hypermethylated in association with faster PLR latency at 24 months. Both genes are critical for neuronal functioning and development; *NR4A2* is implicated in dopaminergic neural cell differentiation^40^, and *HNRNPU* is linked to synaptic function^41^ and neurogenesis^42^. Further supporting the relevance of these findings, gene ontology (GO) enrichment analysis revealed that differentially methylated probes associated with all PLR latency phenotypes, except for cross-sectional 24-month, were significantly enriched for neurodevelopmental processes. These included neurogenesis (GO:0022008), nervous system development (GO:0007399), negative regulation of developmental processes (GO:0051093), and regulation of cellular component organisation (GO:0051128). These findings are notable given previous evidence pointing to the contributing role of DNAm associated with differences in neurodevelopmental processes linked to autism^18^. Collectively, the current findings support PLR latency as a candidate intermediate phenotype for autism, and highlight the association of differential DNAm (and potentially implicated neurodevelopmental processes) to the previously documented associations between PLR latency development and early-emerging autism characteristics^5,13,14^.

At a discovery threshold, differential DNAm of multiple probes significantly associated with PLR amplitude, as did several regions. PLR amplitude has previously been linked to autism^5,13,14^, and although no discovery significant probes nor probes within significantly associated regions were found to have direct associations with autism according to the EWAS catalogue^29^, 12 genes annotated with amplitude-associated probes were in the SFARI database^30^. Nine of these were categorised as ‘strong candidates’ for autism. These autism-associated genes reiterate the diversity of the biological pathways connected to PLR amplitude highlighted in the GO analysis, with gene functions ranging from immunity (*CARD11)*^40^, to metabolic pathways and circadian regulation (*CSNK2B*)^40,43^, to glycoprotein production (*GALNT10)*^40^, and calcium signalling pathways (*CACNA1D*)^40^. Several of the identified autism-associated genes linked to amplitude also play a role in neurodevelopment^40^. These findings add to previous work^5,13,14^ to provide further support for PLR as a candidate intermediate phenotype for autism, and suggest the involvement of differential DNAm across a relatively broad network of genes in infant PLR amplitude variability^5,13,14^.

Our findings must be interpreted in the light of certain limitations. The sample was relatively homogeneous; all participants were male, DNA methylation and phenotypic measures were collected within narrow developmental windows, and the sample was enriched for familial autism likelihood. This limits the generalisability to females, other developmental stages and individuals with different autism likelihoods (e.g., syndromic autism). Nonetheless, within this restricted sample, these findings may inform research on early neurodevelopment and the design/implementation of targeted supportive strategies, though broader application should be approached cautiously and requires further validation. Studying DNAm in a sample of children of the same sex and with DNA and phenotypic measures collected within a tight timeframe likely enhances power by reducing variability unrelated to PLR and associated DNA methylation, and improving signal-to-noise ratios^28,44^. Supporting this, although the sample size was relatively small, four epigenome-wide significantly associated probes were identified. Larger cohorts will be valuable for uncovering additional effects and further elucidating the interplay between epigenetics, PLR and later neurodevelopmental outcomes. Future studies could build on these findings by integrating parent-report and behavioural data to examine how PLR-related epigenetic variation relates to emerging developmental and behavioural profiles over time. While such data were available for the broader cohort, we did not include developmental or familial-likelihood measures as covariates in the current analysis because doing so would risk controlling for variability that is theoretically central to the PLR signal of interest. Our previous work in this sample has already characterised associations between PLR and later developmental outcomes^5^, but meaningfully incorporating epigenetic variation into those models would require substantially larger samples. Larger, well-powered cohorts will therefore be needed to test whether epigenetic variation mediates or moderates links between PLR and later autism-related development.

A second potential limitation is that quantifying DNAm in the PLR reflex neurocircuitry tissue, instead of buccal cells, may yield more specific results related to PLR. However, buccal cells, being more accessible, allow for a larger sample size from live individuals that are deeply phenotyped over time (like our current sample), and are more practical and accessible than post-mortem brain tissue. Further, empirical evidence shows that DNAm patterns in various brain regions are more similar to saliva samples (which contain many buccal cells) than blood samples^45^, and we accounted for variance attributable to differences in cell type proportions by including the proportion of common cell types in buccal samples as a covariate in our analysis.

Another limitation is that while the Illumina 450k array is a reliable tool for quantifying numerous variable DNAm sites, it only covers 2% of all DNAm sites across the genome^46^. Limited coverage may hinder conclusions as probes related to PLR or autism may be unmeasured. Additionally, although we aimed to explore the association of PLR with early altered DNAm (at 9 months), this window for observing the role of DNAm in regulating PLR is narrow. Investigating DNAm at other timepoints or longitudinal changes in DNAm across infancy may offer complementary insights. Finally, while our study provides an initial in silico assessment of autism relevance, the MRC-IEU EWAS Catalogue and SFARI Gene database are continuously expanding resources; therefore, the absence of overlap between our findings and these databases should be interpreted with caution. Replication using other available or emerging datasets could strengthen confidence in the observed associations and clarify their relevance to autism.

In conclusion, this study is, to our knowledge, the first to investigate the association of DNA methylation with the infant pupillary light reflex (PLR), an early-emerging candidate neurophysiological intermediate phenotype associated with autism. We identified four differentially methylated probes significantly associated with PLR latency phenotypes between 14 and 24 months of age, surpassing stringent epigenome-wide significance thresholds (*p* < 2.4 × 10⁻⁷). In addition, 13 differentially methylated regions (DMRs) were associated with PLR latency, particularly with developmental changes occurring over the 14- to 24-month period. While no probes reached epigenome-wide significance for PLR amplitude, several were associated at the discovery threshold (*p* < 5 × 10⁻⁵), along with multiple DMRs, some annotated to genes previously associated with autism. Gene ontology and pathway analyses implicated nervous system development and neurobiological processes in both latency and amplitude phenotypes, with results suggesting a more complex regulatory basis for PLR amplitude.

Together, our study findings not only shed light on the distinct biological underpinnings of early PLR latency and amplitude variability but also highlight the importance of considering both biological specificity and developmental timing when evaluating epigenetic contributions to early neurodevelopment. This work opens a new line of inquiry into how epigenetic signatures associate with neurophysiological processes in infancy - rather than higher-order cognitive processes - might forecast neurodevelopmental outcomes. By identifying methylation patterns linked to this reflex, we move closer to understanding individual differences in sensory-motor and autonomic regulation that have previously been associated with early autism, thereby contributing to a more nuanced understanding of neurodevelopmental diversity.

Further, support or services aimed at promoting well-being are often most effective when implemented during infancy, a period of high neurodevelopmental plasticity^47^. Insights from the current research may help guide the identification of individuals who could benefit most from early support, and/or inform the design of such support - particularly those addressing sensory, motor, or autonomic differences that some autistic individuals or their families report as highly impactful on daily life^48–50^. In this way, our findings contribute to a foundation for future research to explore biologically informed approaches that prioritise the well-being of neurodivergent individuals, as well as advance understanding of the biological diversity underlying early development.

## Methodology

### Sample

Participants in the current analysis were recruited into the British Autism Study of Infant Siblings (BASIS; www.basisnetwork.org; SM 1), a longitudinal study of the development of infants with familial autism likelihood due to having a first degree autistic relative. Full ethical approval was granted by NHS Research Ethics Committees (08/H0718/76 [BASIS] and 06/MRE02/73 [DNA collection, extraction and analysis]). All methods were performed in accordance with NHS research governance frameworks and relevant international ethical standards, including the Declaration of Helsinki. Informed written consent was provided by the parent(s).

The current sample consisted of a total 51 male infants who had DNA methylation data quantified at 9-months, and PLR data from at least one of the following time points: 9-months, 14-months or 24-months. The sample was enriched for infants with familial autism likelihood (*N* = 42); 9 infants had no familial autism likelihood, having no family history of autism. Including infants with and without a familial likelihood for autism introduced a modest degree of phenotypic heterogeneity, which was advantageous for capturing broader, but still relevant, variability in PLR to better assess associated epigenetic variation in this candidate intermediate phenotype.

We focused specifically on these age windows of PLR measurement because *(1)* previous investigations have reported an association of the PLR change across this period with a later autism diagnosis, and *(2)* this is the key early developmental timeframe in which behavioural autism characteristics gradually emerge^51^. As DNAm is dynamic over development^18^, we focused on DNAm at our first PLR timepoint (9 months) to investigate the developmental sequelae in PLR following earlier DNAm and to narrow the age range of DNAm as recommended by previous investigations^52^. We additionally included only male infants to minimise epigenetic heterogeneity related to sex.

### Pupillary light reflex (PLR)

The PLR was induced while infants passively watched stimuli that transitioned from a black to white screen on a monitor^53^. Stimulus was presented to the cohort 32 times at 9 and 14 months and 16 times at 24 months (see SM 2 Fig. 1). Pupil diameter data was collected at 9-, 14- and 24-months using Tobii T120 eye tracker (sampling rate = 60 Hz; Tobii Technology AB, Danderyd, Sweden; millimetres to 2 decimal places). Pre-processing of raw pupil data (see SM 2.1.2) removed artefacts and extracted PLR latency (constriction onset time; ms) and amplitude (constriction magnitude relative to average pupil size before latency; %). Latency was defined as the minimum acceleration from 110-570ms after white slide onset. Amplitude was calculated as the maximum constriction within the interval 170–1450ms post-latency, relative to the average pupil diameter size at latency onset^54^. The parameter identification time windows were determined by a series of optimisation investigations (see SM 2.1.1). Three or more valid trials per time point were required for inclusion. The median latency and amplitude per individual per time point were calculated.

To account for the variance in the PLR parameters at each timepoint explained by the covariates of missingness (i.e., percentage of missing trials from the stimuli) and baseline pupil diameter (i.e., the average pupil diameter up to 100ms before PLR latency), we conducted linear multiple regression analysis with latency or amplitude as the dependent variables and missingness and baseline as the predictor variables (SM 2.2). Linear regressions were conducted within timepoint. Residuals were extracted and used in all subsequent analysis as the PLR latency and amplitude variables.

In total 10 PLR phenotypes were analysed in this study. This included 6 cross-sectional PLR measures (latency and amplitude at 9-, 14- and 24-month) and 4 PLR developmental change scores (9- to 14-month and 14- to 24-month for latency and amplitude). Change scores were calculated using subtraction of the older minus the younger measure. The inclusion of all 10 PLR phenotypes, encompassing both cross-sectional and developmental measures, is based on the current literature, which suggests that both latency and amplitude at various time points across infancy may be associated with autism either directly such as 10-month amplitude and latency^13,55^, or as part of an associated developmental trajectory^5,13^. The inclusion of these phenotypes enables a comprehensive exploration of the role of epigenetics in early PLR as a candidate intermediate phenotype for autism.

### DNA methylation (DNAm)

Buccal samples were collected at 9 months from 51 infants and processed using a standard pipeline^28^. Genomic DNA (500ng) from each sample was extracted and treated with sodium bisulfite using the Zymo EZ DNA Methylation-Lightning Kit™ (Zymo Research, Irvine, CA, USA). The Illumina Infinium^®^ HumanMethylation450 BeadChip kit (450 K array; Illumina, San Diego, CA, USA) was used to assess DNA methylation at 482,421 sites (∼2% o all CpG loci) throughout the genome. We then quantified DNAm using the HiScan System (Illumina, San Diego, CA, USA) and extracted signal intensities using Illumina GenomeStudio software (Illumina, San Diego, CA, USA) for each probe.

All epigenomic data processing and downstream analysis was conducted using R version 4.0.2. and R studio^56,57^ on the high-performance computing facilities at King’s College London (CREATE). Methylation signal intensity data was imported using *methylumi R-*package^58^. Data quality control and pre-processing was conducted using *wateRmelon* R-package^59^. The ‘pfilter’ function removed failed samples and probes (e.g., low bead counts (< 3) or detection p-values > 0.05 for more than 1% of probes). The ‘dasen’ function was then used to normalise the data using a standardised pipeline^59^ to address background correction, dye bias, probe adjustments, batch effects, and other non-biological variations related to external parameters (e.g., array order)^60^. Cross-reactive and polymorphic probes, as identified in Illumina annotation files and recent studies^61,62^, were excluded. The final dataset included 402,971 probes with methylation levels expressed as beta (β) values ranging from 0 (unmethylated) to 1 (fully methylated), as shown in SM 3.1 Fig. 1.

### Statistical analysis

#### Epigenome-wide association study (EWAS) analysis

In total, 10 separate EWAS analyses were conducted using linear multiple regressions fitted individually for each CpG. Models were built with processed DNAm β as the dependent variable and the PLR phenotype as the predictor with age, batch, 10 principal components (identified using location-based principal component analysis^63^, SM 3.2) and estimated cell type proportion for 7 common cell-types in buccal samples (identified using EpiDISH R package^64^, SM 3.3) as covariates. To account for account for multiple testing in epigenome-wide association studies (EWAS), we applied a ‘stringent’ 450k *significance* threshold of *p* < 2.4 × 10^− 7^^65^, and a more liberal ‘discovery’ *significance* threshold of *p* < 5 × 10^− 5^, as commonly utilised in EWAS of complex phenotype including traits associated with autism^24,26–28,66^.

#### Differential methylated region (DMR) analysis

We used *dmrff* R-package^67^ to identify DMRs associated with the 10 phenotypes of interest. DMRs were determined using EWAS summary statistics and defined as regions of two or more neighbouring probes within 500 base pairs with EWAS *p-value* < 0.05 and effect estimates in the same direction (negative or positive). The p-value threshold for significant DMR was set at 0.05 after Bonferroni adjustments.

#### Downstream exploratory analysis

To assess the biological significance of genes linked to probes associated with the PLR phenotypes, we performed Gene Ontology (GO) enrichment analysis using the top 1,000 probes (ranked by *p-value*) and significantly associated DMRs (SM 5 Table 1). Analysis focused on biological processes and was conducted with the PANTHER overrepresentation test^68–70^, referencing genes annotated to the 402,971 probes used in EWAS and DMR analyses. Gene annotations were based on Illumina UCSC hg19 annotations^71^. Significant enrichment required at least two genes per term and an FDR-adjusted *p-value* ≤ 0.05. We investigated whether probes significantly associated with PLR in the EWAS or DMR analysis were previously linked to autism in the MRC-IEU EWAS catalogue^29^ (October 2024) or located within autism-associated genes listed in the SFARI Gene database^30^ (September 2024; gene.sfari.org).

## Supplementary Information

Below is the link to the electronic supplementary material.

Supplementary Material 1

Supplementary Material 2

## Data Availability

The datasets analysed during the current study are not publicly available but can be requested from the Emily J. H. Jones (a co-author of this manuscript and author of the original studies).
