# Supplementary material for: Epigenome-wide analysis identifies DNA methylation signatures associated with the infant pupillary light reflex, a candidate intermediate phenotype for autism
Source: Sci Rep. 2026 Jan 8;16:325. doi: 10.1038/s41598-025-31651-5 (PMC12783723; doi:10.1038/s41598-025-31651-5)
Supplement: Supplementary file 2 — Supplementary Material 2 [file 41598_2025_31651_MOESM2_ESM.docx]

**Supplementary Material**

Table of Contents

[SM 1: Study cohort and procedure 3](#_Toc216192355)

[SM 2: PLR processing and covariates 5](#_Toc216192356)

[SM 2.1: PLR Processing pipeline 5](#_Toc216192357)

[SM 2.1.1: PLR Processing optimisation 5](#_Toc216192358)

[SM 2.2 PLR covariates 10](#_Toc216192359)

[SM 2.2.1 Missingness 10](#_Toc216192360)

[SM 2.2.2 Baseline pupil size 10](#_Toc216192361)

[SM 2.2.3. Accounting for covariates in PLR variables 11](#_Toc216192362)

[SM 3: DNA Methylation data processing 12](#_Toc216192363)

[SM 3.1. DNA Methylation level 12](#_Toc216192364)

[SM 3.2: Location-based principal component for Epigenome-wide Analysis (EWAS) 12](#_Toc216192365)

[SM 3.3: Cell-type principal component for Epigenome-wide Analysis (EWAS) 13](#_Toc216192366)

[SM 4: EWAS results 14](#_Toc216192367)

[SM 4.1 Lambda inflation factor Q-Q plots and EWAS Manhattan plots for each EWAS 14](#_Toc216192368)

[SM 4.2: EWAS probes at discovery p-value threshold (p < 5 × 10^-5^) or above. 17](#_Toc216192369)

[SM 4.3: Significantly associated DMR probes 17](#_Toc216192370)

[SM 5: Downstream exploratory analysis. 19](#_Toc216192371)

[SM 5.1: Gene Ontology input 19](#_Toc216192372)

[SM 5.2: Gene Ontology results 19](#_Toc216192373)

[SM 5.3: SFARI gene comparison 19](#_Toc216192374)

[References 20](#_Toc216192375)

# SM 1: Study cohort and procedure

The British Autism Study of Infant Siblings (BASIS; [www.basisnetwork.org](http://www.basisnetwork.org/)) is a prospective longitudinal study aiming to track the emergence of early autism characteristics in a sample of children with about 20% increased likelihood for receiving a later autism diagnosis due to having a first-degree relative with a diagnosis of autism^1^. Measurements are taken at 9 months, 14 months, 24 months and 3 years using a battery of parent-report questionnaires, eye-tracking and EEG experimental tasks, standardised behavioural assessments and clinical assessments. Infants were born full-term (36+ weeks) and had no known genetic syndromes or visual, auditory, or other disabilities.

The sample for the current paper participated in the second phase of the BASIS and had buccal samples taken for epigenetic investigations at 9 months. SM 1 Table 1 summarises the sample characteristics.

SM 1 Table 1. Summary of sample for each EWAS including total number of participants (and number of those with increased and low without familial autism likelihood for autism), average age and average PLR measure.

| Phenotype | N  (FL, NL)^1^ | Age in months  Mean (SD) | PLR  Mean (SD)^2^ |
| --- | --- | --- | --- |
| **Latency (ms)** |  |  |  |
| 9 months | 48  (40, 8) | 9.07  (0.85) | 329.18 (40.05) |
| 14 months | 47  (38, 9) | 15.44  (1.02) | 317.54 (41.05) |
| 24 months | 40  (32, 8) | 26.22  (2.04) | 312.41 (29.38) |
| 9 to 14 months | 44  (36, 8) | - | -15.45 (35.56) |
| 14 to 24 months | 37  (29, 8) | - | 0.93  (39.78) |
| **Amplitude (%)** |  |  |  |
| 9 months | 46  (38, 8) | 9.06  (0.85) | 32.89  (7.71) |
| 14 months | 47  (38, 9) | 15.44  (1.02) | 36.16  (8.27) |
| 24 months | 37  (29, 8) | 26.15  (2.09) | 28.12  (7.81) |
| 9 to 14 months | 42  (34, 8) | - | 2.90  (7.21) |
| 14 to 24 months | 34  (26, 8) | - | -8.32  (8.81) |

*Note: ^1^Sample included in EWAS split by autism familial likelihood (NL = No familial autism likelihood; FL = familial autism likelihood). ^2^PLR values are the raw (non-residual) values. There was minimal participant attrition over time (one participant did not complete the 14-month visit, and two the 24-month visit). All other PLR data loss across the sample was due to individual trials not meeting the ≥3 valid trial threshold required for processing and analysis (see SM 2.1.2.). This mostly affected the 24-month pupil data, which included 16 stimulus trials compared with 32 stimulus trials at 9 and 14 months, resulting in more instances where infants did not reach the valid trial threshold. Additionally, both timepoints in a change score were required to meet this threshold, leading to more missing data points in the change scores than in the cross-sectional scores.*

# SM 2: PLR processing and covariates

## SM 2.1: PLR Processing pipeline

## SM 2.1.1: PLR Processing optimisation

The PLR processing pipeline implement is the same as that described previously ^2,3^ with aspects of the pipeline optimised using observations of the current dataset, for parameter identification (e.g. latency and maximum constriction window) and attrition (e.g. later exclusion criterion).

To optimise the identification of PLR latency and amplitude parameters, an initial PLR processing pipeline ^2^ was systematically evaluated and refined. In the original configuration of the pipeline, which was informed by empirical visualisation of an infant PLR dataset, latency was defined as the point of maximum pupil acceleration within a fixed 100–400 ms window after stimulus onset, and maximum constriction was defined as the minimum pupil diameter within a 500–1500 ms window. However, with the current dataset, when using the original pipeline, an unexpectedly high number of latency values occurred at the upper boundary of the 100–400 ms window, indicating that these time constraints for latency were too restrictive. The pipeline was therefore re-run without time restrictions to empirically determine optimal latency and constriction windows. Latency was thus defined as the point of maximum acceleration between stimulus onset and the minimum pupil diameter (0–2500ms), and maximum constriction as the minimum pupil size between latency and 2500ms. Manual visualisation and validations of the data from this unrestricted processing were conducted using TimeStudio^4^. This identified the 0.1–99.9th percentile range of true response times, resulting in an optimised latency window of 110–570ms post-stimulus and a maximum constriction window of 170–1450ms relative to latency. Additionally, improved visualisation tools were introduced to enhance manual validation accuracy by flagging values at window boundaries. These optimisations were piloted on 30% of the sample, demonstrating improved distributional properties and reduced skewness in extracted parameters compared to the original processing pipeline.

To reduce data attrition, the exclusion criteria set in the original^2^ processing pipeline were evaluated and refined. The original exclusions proved overly strict for the current dataset, likely because all pupil data was recorded using a higher frequency eye-tracker (Tobii T120 eye tracker; 60Hz) than in ~30% of the data points in the original Nyström et al. (2018; Tobii 1750 eye tracker; 50 Hz) dataset. In particular, the criterion excluding trials where the latency or maximum constriction time fell on or adjacent to interpolated data points disproportionately affected trials from the higher-frequency eye-tracker. This rule was therefore modified to exclude only those trials where 100% of the data within ±40 ms of the latency or constriction point (equivalent to the 25-point moving average window applied to 300 Hz resampled data, see below) were interpolated or missing. This adjustment substantially reduced participant and trial attrition while maintaining the integrity and efficiency of the automated processing pipeline.

**SM 2.1.2: PLR Processing**

Pupils were measured to be at an average distance of 59.54 cm (59.15 cm–59.95 cm) during the PLR. Following previous work^2^, to control for differing PLR inducing light levels across the white slide in stimulus one, only trials where the first look was within the centre 5% of the white slide (see SM 2 Figure 1) were included. First, an automated cleaning algorithm was applied to data using R ^5^ and R-studio ^6^. This algorithm involved: the removal of measurement outside the range of 1-10mm diameters; linear interpolation of short “flicker” gaps (<7 samples) with <0.2mm diameter change; removal of samples with large diameter changes from the previous sample (>0.3mm); removal of short segments of data (<6 samples); additional linear interpolation; resampling data to 300hz ^7^ and application of a 25-point moving average filter ^2^ supported by visual inspection of data. This cleaning algorithm resulted in a continuous smoothed pupil trace that, compared to the raw pupil trace, had a higher resolution and fewer potential artefacts. Using this pupil trace, we calculated first (velocity) and second (acceleration) order derivatives, while applying 25-point moving average filters between each derivation to reduce noise amplification ^7^. Time windows for minimum and maximum constriction were determined by a series of optimisations investigations. The PLR latency was defined by the acceleration minima in the time interval 110-570ms to the stimuli onset. The baseline pupil size for each trial was defined as the average pupil size in a 100ms interval just before the latency time point. The amplitude of the PLR was calculated using the formula presented in SM 2 Figure 1, using the maximum constriction within the interval 170– 1450ms relative to latency onset.

**SM 2 Figure 1. PLR stimulus and pupil response**. a) Stimulus presented to the cohort, 32 times at 9/14 months and 16 times at 24 months; b) PLR inducing stimulus slide with blue circle demonstrating the approximate location of the centre area of interest (centre ~5% of the white slide) for first look for trial to be included in the analysis; c) pupil size (mm) and acceleration (a.u.) traces demonstrating relative constriction amplitude formula components ^8^*(A*_0_ = average pupil diameter in the window 100ms before constriction onset latency; *A*_m_ = minimum pupil diameter in 170-1450ms relative to *A_0_*. ms = milliseconds).

All pupil traces were visually inspected for manual validation and were rejected based on two criteria: a) just the latency was incorrectly identified or b) both the latency and the maximum constriction was incorrectly identified. For example, if a pupil trace had a valid latency but invalid maximum constriction (e.g. the infant blinked after the PLR latency, but before maximum constriction), then the trace was accepted only for its valid latency and included in latency analysis only (not amplitude analysis). We conducted inter-rater reliability checks on the manual validation. A second independent validator scored 20% of trials. Unweighted Cohen’s Kappa Coefficient was used to assess the agreement between two manual validators’ judgement on whether PLR processing across the whole all measurement from the BASIS sample (phase 2 and phase 3) correctly extracted PLR latency or the maximum constriction (latency: κ = .86 [95% CI, .84 to .88], p < .001, constriction: κ = .84 [95% CI, .82 to .86], p < .001).

Once manual validation had been completed, those trials where either latency or both latency and maximum constriction points had been successfully identified were passed to the second pipeline of automated processing. For each trial, the eye (left or right) with the pupil trace that best correlated (parallel correlations according to p-value and correlation coefficient) with all other valid pupil traces for that participant at that assessment timepoint were extracted. If neither the left nor right eye’s trace significantly correlated with the remaining manually validated traces, then that trial was excluded.

Trials were excluded if: a) the latency window had more than 75% interpolated or missing data in original trace; b) the maximum constriction window had more than 75% interpolated or missing data in original trace; c) the amplitude was outside a range of 5% to 80% (thus biologically implausible); d) 100% of the window +/-40ms relative to latency or maximum constriction was interpolated data (+/-40ms being the size of the 25pt moving average window around the latency/maximum constriction point). Participants with fewer than 3 trials remaining after processing were excluded. The median latency and amplitude per individual per timepoint were calculated and included in models as dependent variables. The averaging of PLR parameters was conducted in line with findings ^7^ whereby authors concluded that for 60-Hz sampling rate, averaging latencies of multiple pupil light reflexes helped to improve the resolution of latency because the average latency was not constrained to a time grid of 16.7ms.

## SM 2.2 PLR covariates

We considered the trials’ missingness (SM 2.2.1) and baseline pupil size (SM 2.2.2) as a potential covariate for our PLR measures.

### SM 2.2.1 Missingness

The PLR, especially latency, is susceptible to the number of trials included in the averagingClick or tap here to enter text.. Trials could be missing either due to children not watching the stimuli (e.g., if they were distracted or inattentive during the protocol) or were excluded during PLR processing due to poor data quality. We calculated missingness (see SM 2 Table 1) as a percentage of the total number of possible trials in the stimuli (32 for 9/14 months, 16 for 24 months). Missingness was averaged across trials within participants for each time point.

SM 2 Table 1. Mean percentage of trials missing during processing for each PLR parameter at each time point.

|  | Missing (%)  Mean (SD) | | |
| --- | --- | --- | --- |
|  | 9 months | 14 months | 24 months |
| Latency | 59.51 (13.54) | 58.98 (14.05) | 47.81 (20.73) |
| Amplitude | 62.09 (13.2) | 62.77 (14.33) | 55.41 (20.1) |

*Note: values are an average of individual’s missingness percentages. SD = standard deviation*

### SM 2.2.2 Baseline pupil size

The size and strength of the PLR is potentially influenced by the size of pupil prior to the PLR^9^. We calculated baseline pupil size (see SM 2 Table 2) for each trial as the average pupil diameter between 100ms before PLR latency and the PLR latency. Baseline pupil size was averaged across trials within participants for each time point.

SM 2 Table 2. Mean baseline pupil for each PLR parameter for each timepoint.

|  | Baseline pupil size (mm)  Mean (SD) | | |
| --- | --- | --- | --- |
|  | 9 months | 14 months | 24 months |
| Latency | 3.56 (0.41) | 3.62 (0.41) | 3.66 (0.39) |
| Amplitude | 3.55 (0.41) | 3.63 (0.41) | 3.69 (0.36) |

*Note: values are an average of individual’s baseline. SD = standard deviation*

### SM 2.2.3. Accounting for covariates in PLR variables

To account for the variance in the PLR parameters (at each time point: 9, 14, and 24 months) attributable to the covariates, we conducted linear multiple regression models with PLR (latency or amplitude) as the dependent variable and covariates (missingness and baseline pupil size) as the predictor variables. Residuals from these analyses were extracted and used in all subsequent analyses as the PLR variables.

# SM 3: DNA Methylation data processing

## SM 3.1. DNA Methylation level

Processing of DNA methylation resulted in 402,971 probes with methylation level data at each probe expressed as a 'beta' value (β) ranging from 0 (no methylation) to 1 (complete methylation) as illustrated in SM 3 Figure 1.

SM 3 Figure 1. Density plot of the distribution of β-values across sample.

## SM 3.2: Location-based principal component for Epigenome-wide Analysis (EWAS)

To account for sources of unwanted variance attributable to population stratification in the processed DNAm beta values, we conducted a location-based principal components analysis and included the outputted 10 principal components as covariates in the EWAS model. This location-based principal component analysis^10^ was computed using CpG sites within 50 base pairs of Single Nucleotide Polymorphisms (SNPs) with minor allele frequency > 0.01 reported in the 1000 Genomes Project ^11^. Restricting the PCA to CpG sites located near such SNPs narrows the focus of methylation variants that are better proxies of ancestry^10^.

## SM 3.3: Cell-type principal component for Epigenome-wide Analysis (EWAS)

To account for unwanted variance arising from cell-type signals in the processed DNA methylation beta values, we performed an analysis using EPiDISH^12^. This analysis estimates methylation levels for nine common cell types found in buccal samples, allowing us to incorporate these estimates as covariates in our epigenome-wide association study (EWAS) model. Cell types included epithelial cells, fibroblasts, B cells, CD4+T cells, monocytes, eosinophils, neutrophils, CD8+ T cells, natural killer cells.

In our EWAS model, we included all estimated cell-type proportion as covariates, except for CD8+ T cells, which exhibited zero variance, and natural killer cells, which caused singularity in the EWAS model as it was highly correlated with B cell proportion (r = 0.98, p <0.001).

Including the estimated proportion for these cells improved model robustness and confounding effects related to cell-type composition of the buccal samples.

# SM 4: EWAS results

## SM 4.1 Lambda inflation factor Q-Q plots and EWAS Manhattan plots for each EWAS


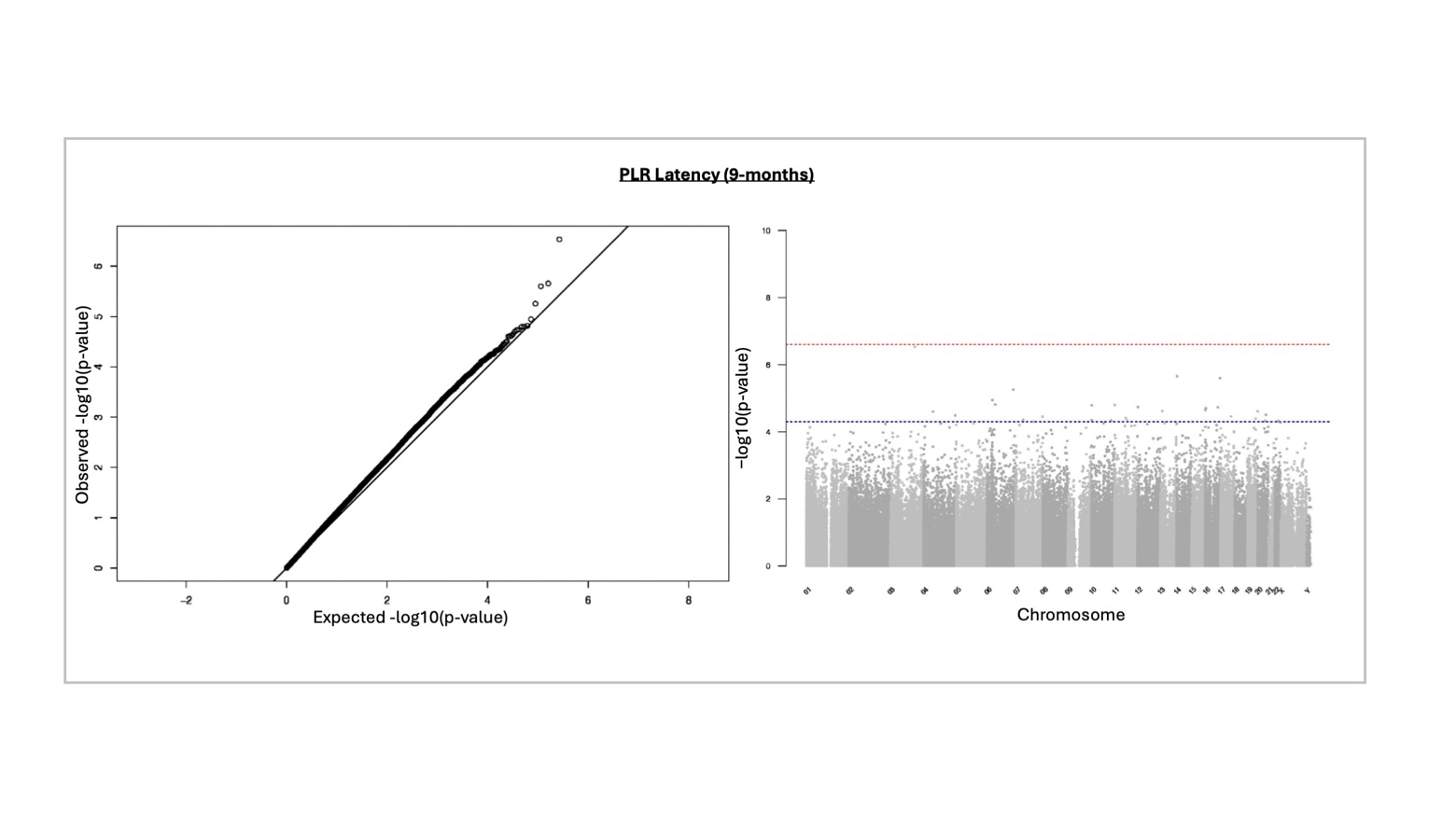


SM 4 Figure 1. Q-Q plot (left) and Manhattan plot (right) of results from 9-month PLR latency EWAS


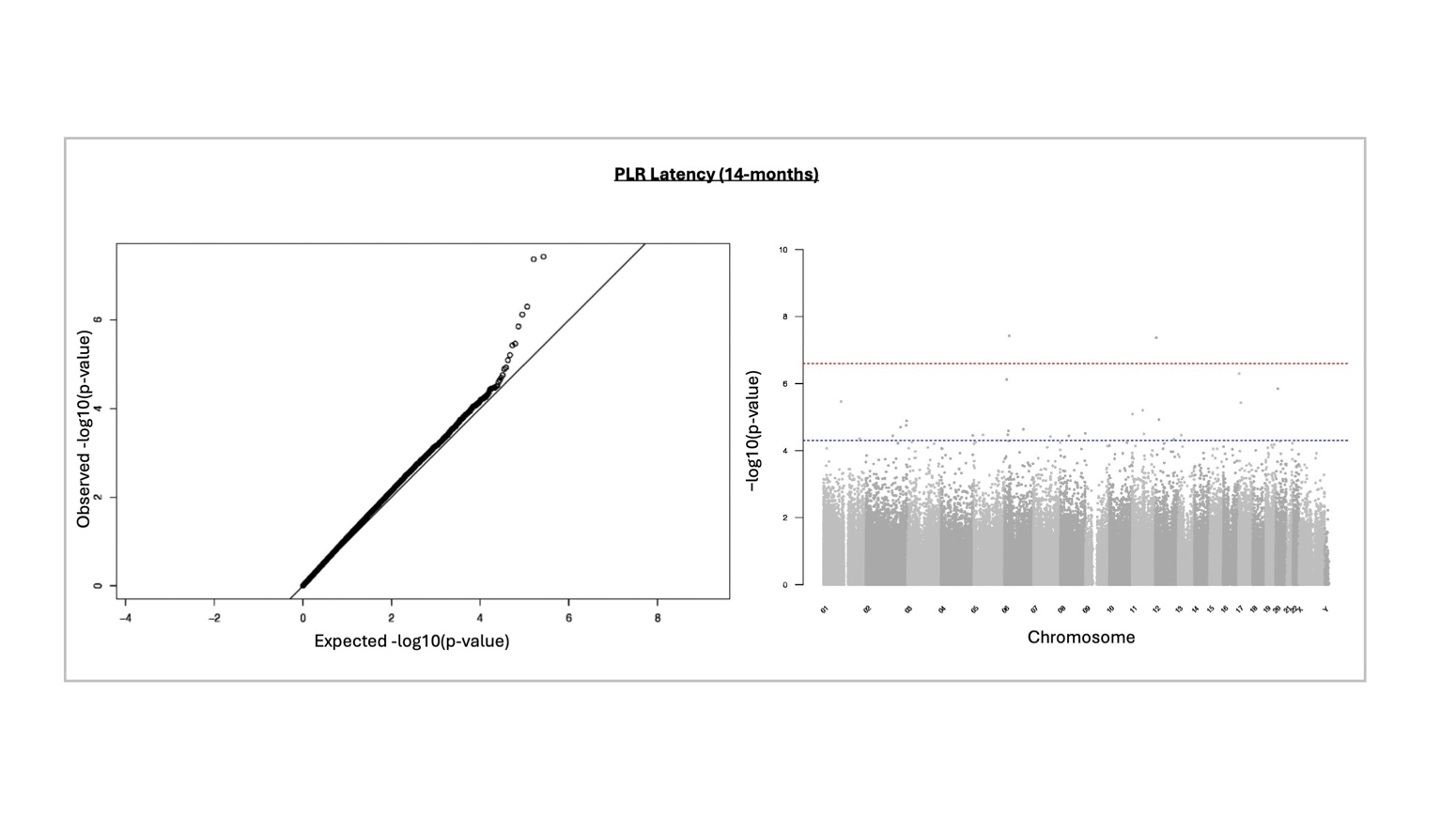


SM 4 Figure 2. Q-Q plot (left) and Manhattan plot (right) of results from 14-month PLR latency EWAS

**
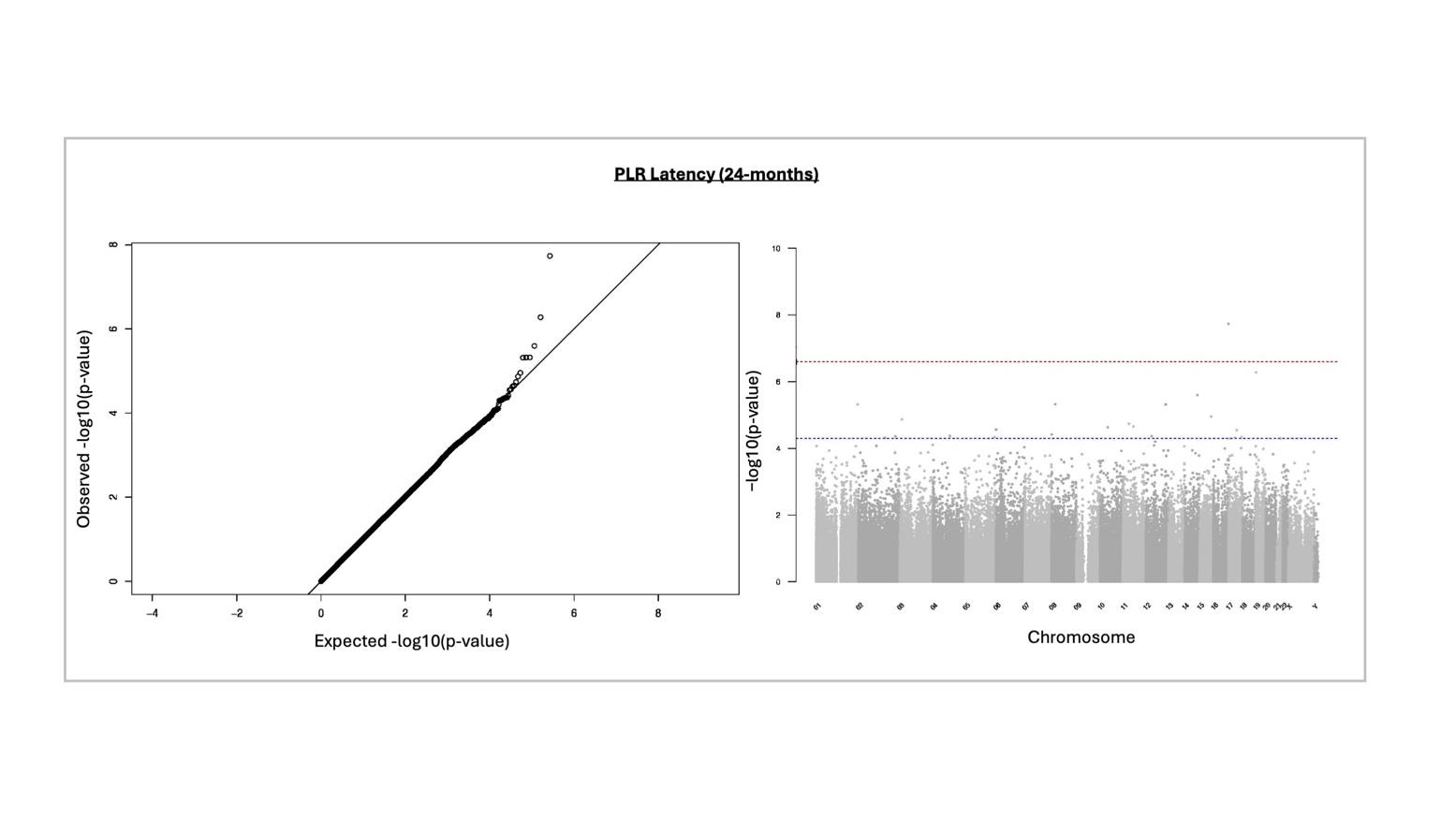
**

SM 4 Figure 3. Q-Q plot (left) and Manhattan plot (right) of results from 24-month PLR latency EWAS

**
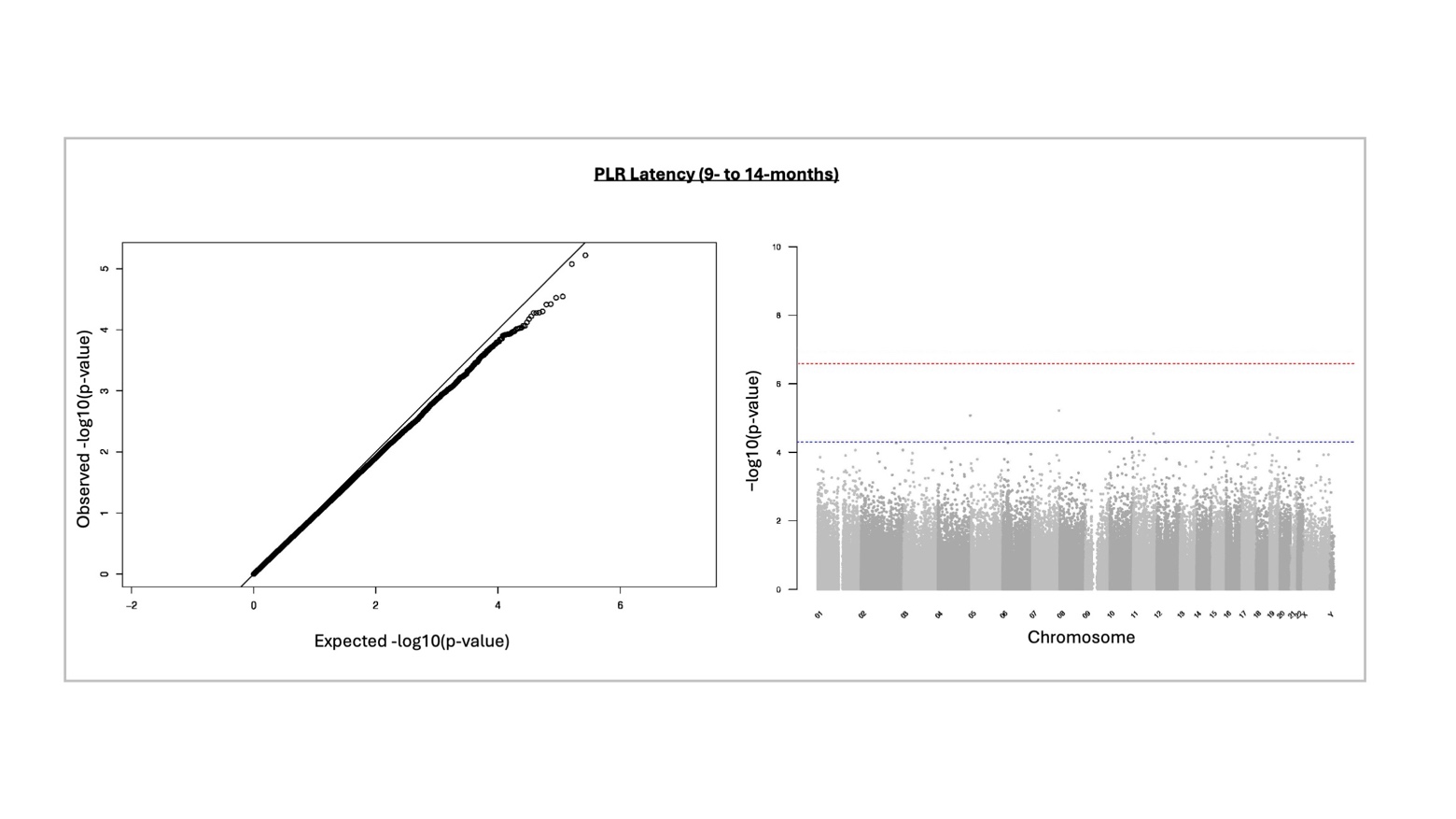
**

SM 4 Figure 4. Q-Q plot (left) and Manhattan plot (right) of results from 9- to 14-month PLR latency EWAS

**
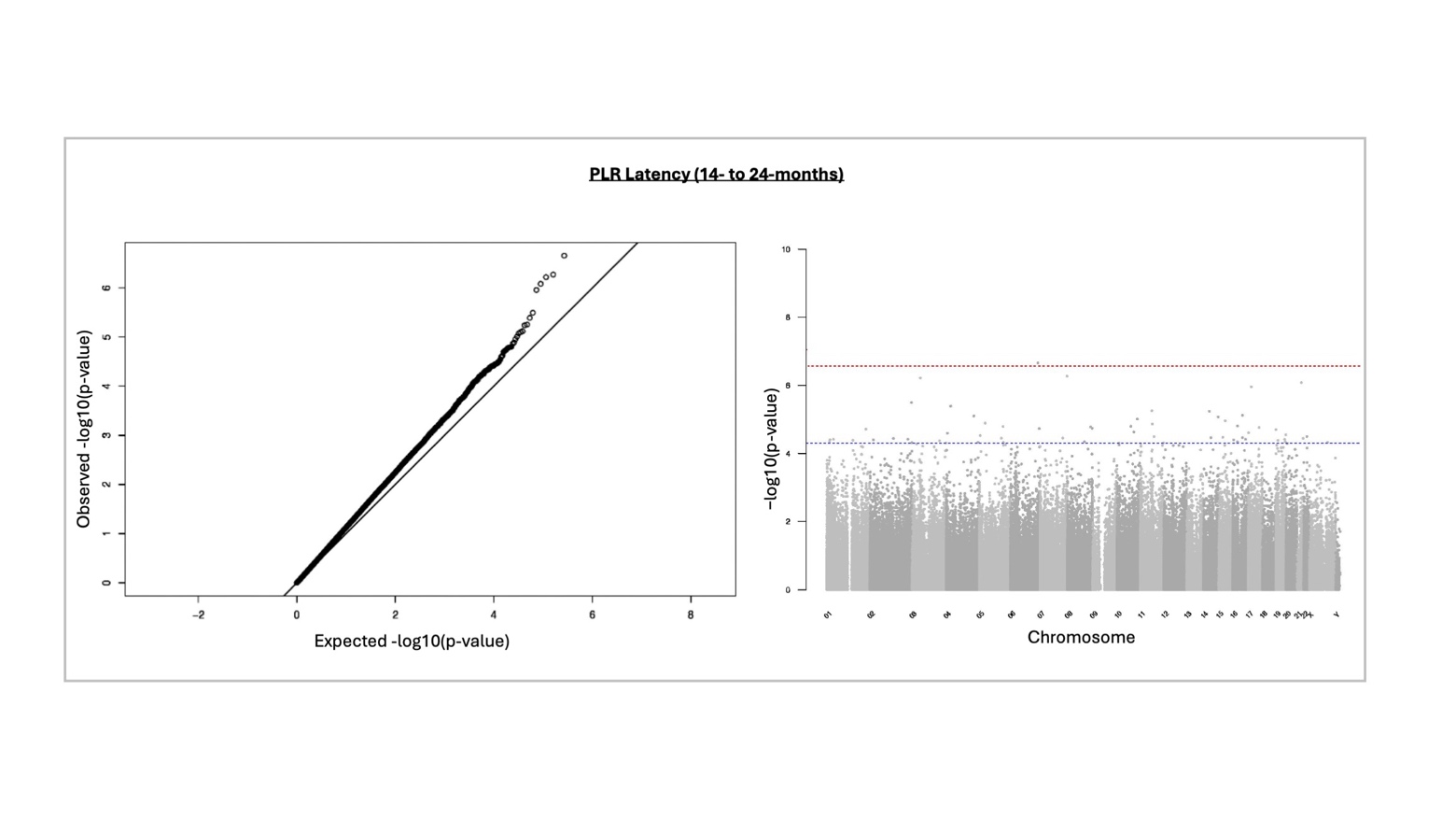
**

SM 4 Figure 5. Q-Q plot (left) and Manhattan plot (right) of results from 14- to 24-month PLR latency EWAS

**
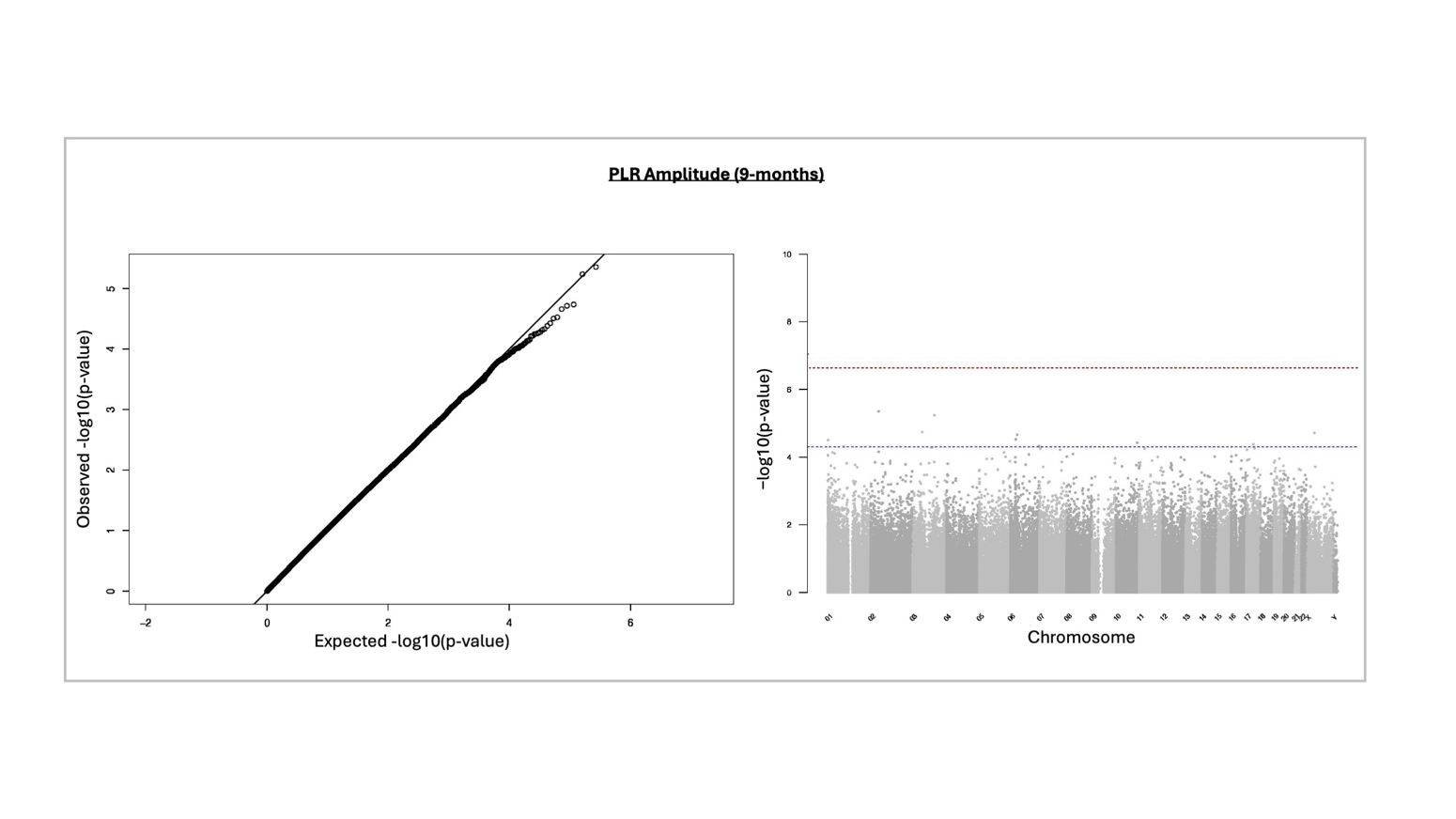
**

SM 4 Figure 6. Q-Q plot (left) and Manhattan plot (right) of results from 9-month PLR amplitude EWAS


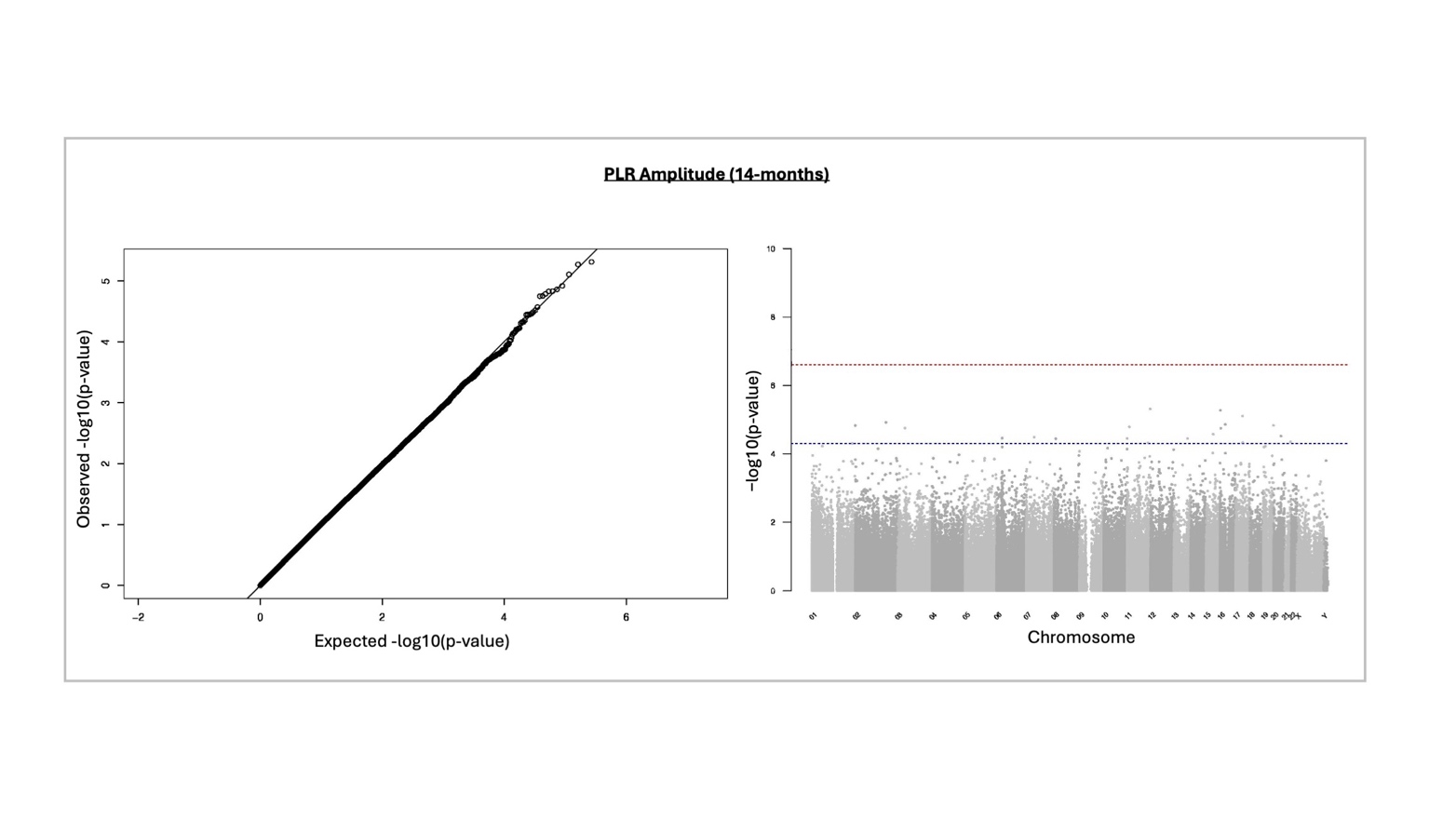


SM 4 Figure 7. Q-Q plot (left) and Manhattan plot (right) of results from 14-month PLR amplitude EWAS

**
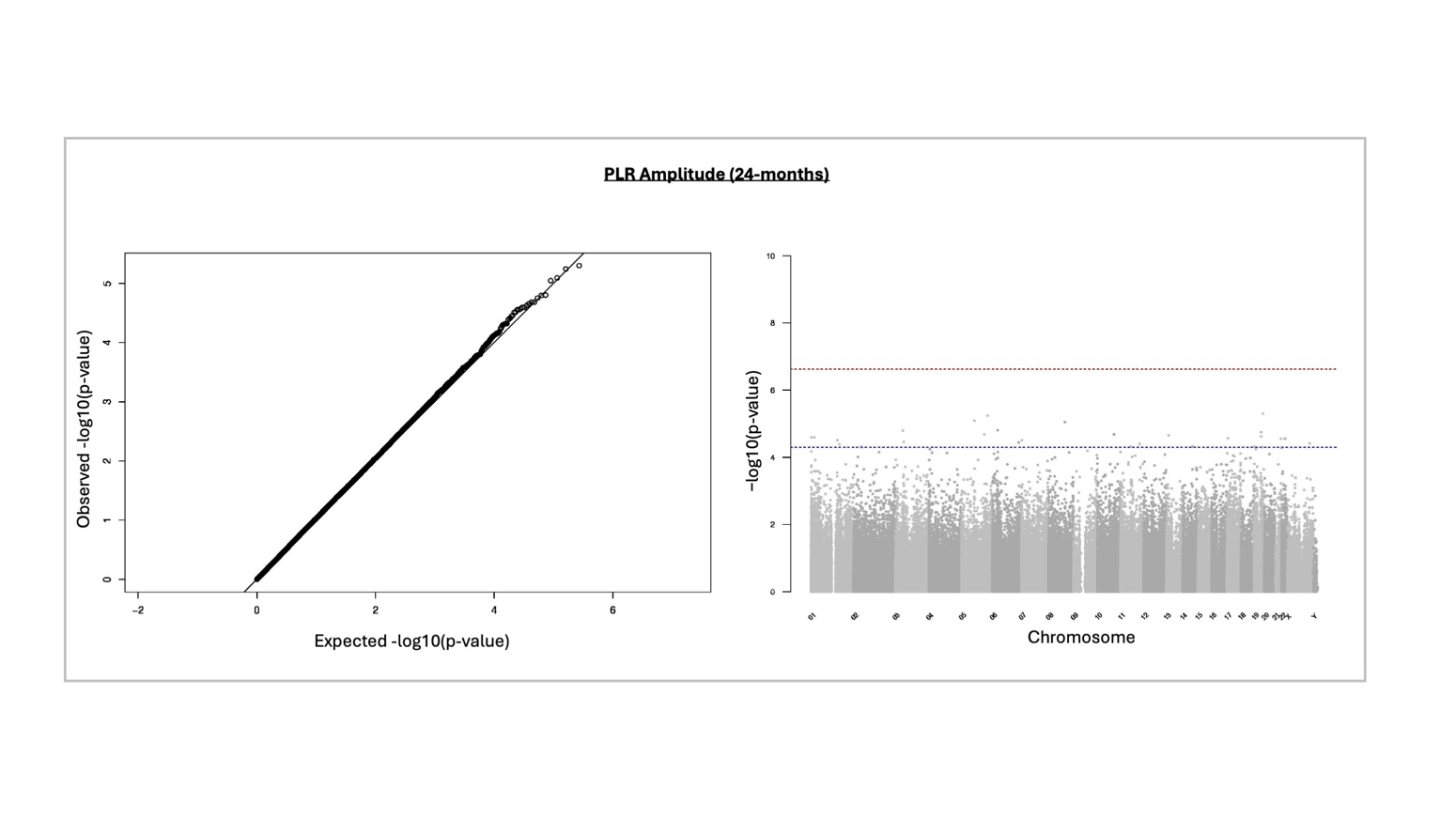
**

SM 4 Figure 8. Q-Q plot (left) and Manhattan plot (right) of results from 24-month PLR amplitude EWAS


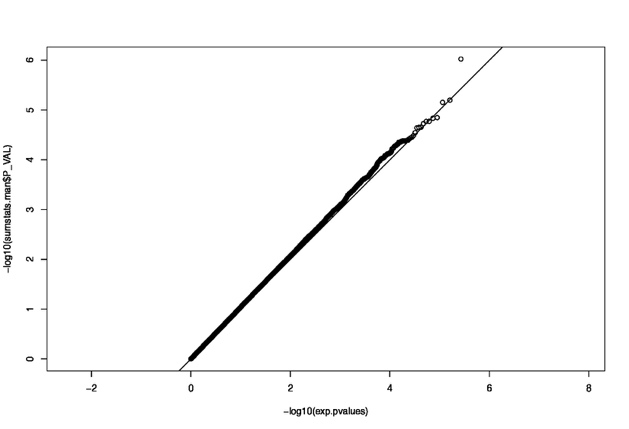

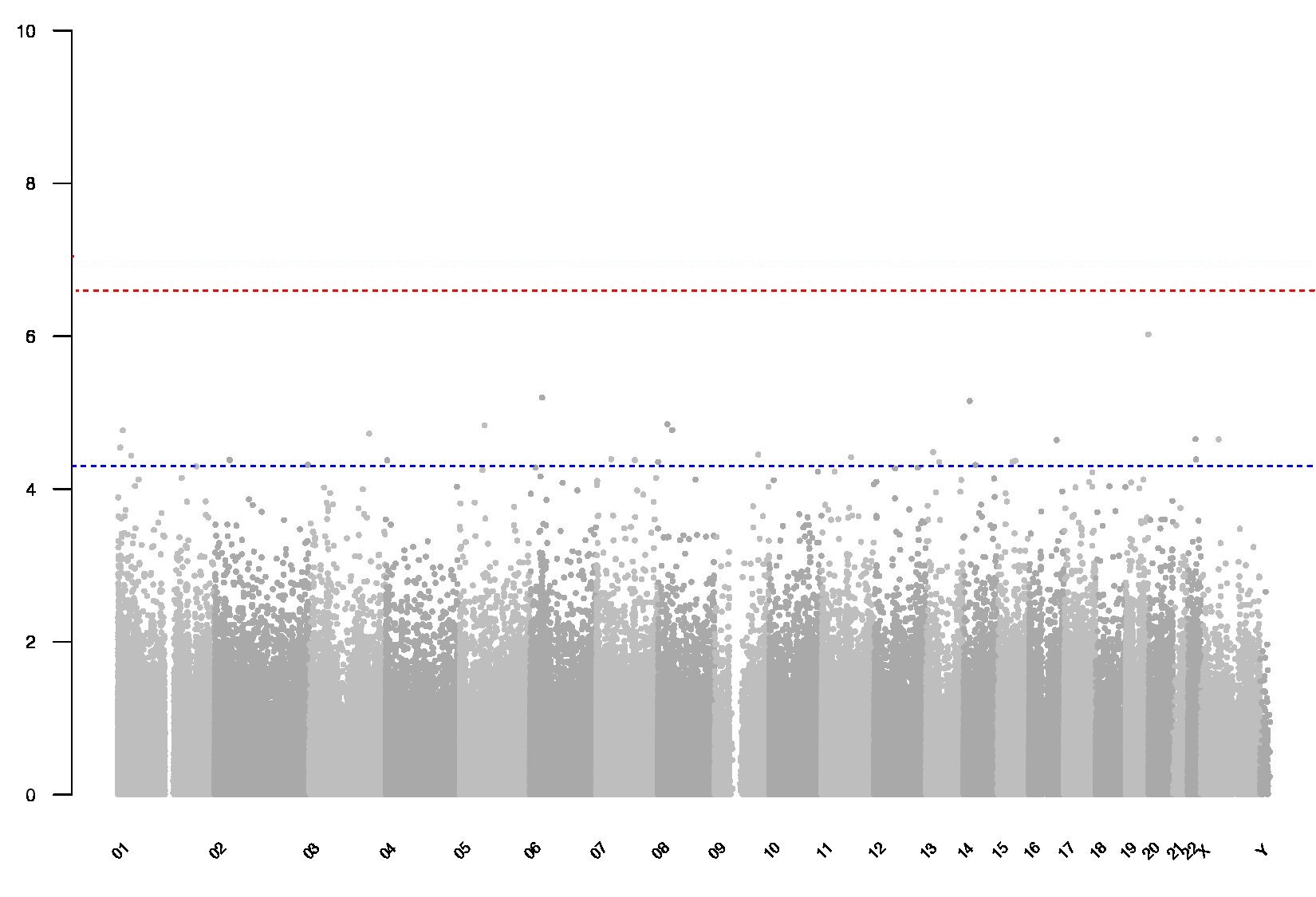


**PLR Amplitude 9- to 14-months**

SM 4 Figure 9. Q-Q plot (left) and Manhattan plot (right) of results from 9- to14-month PLR amplitude EWAS

**
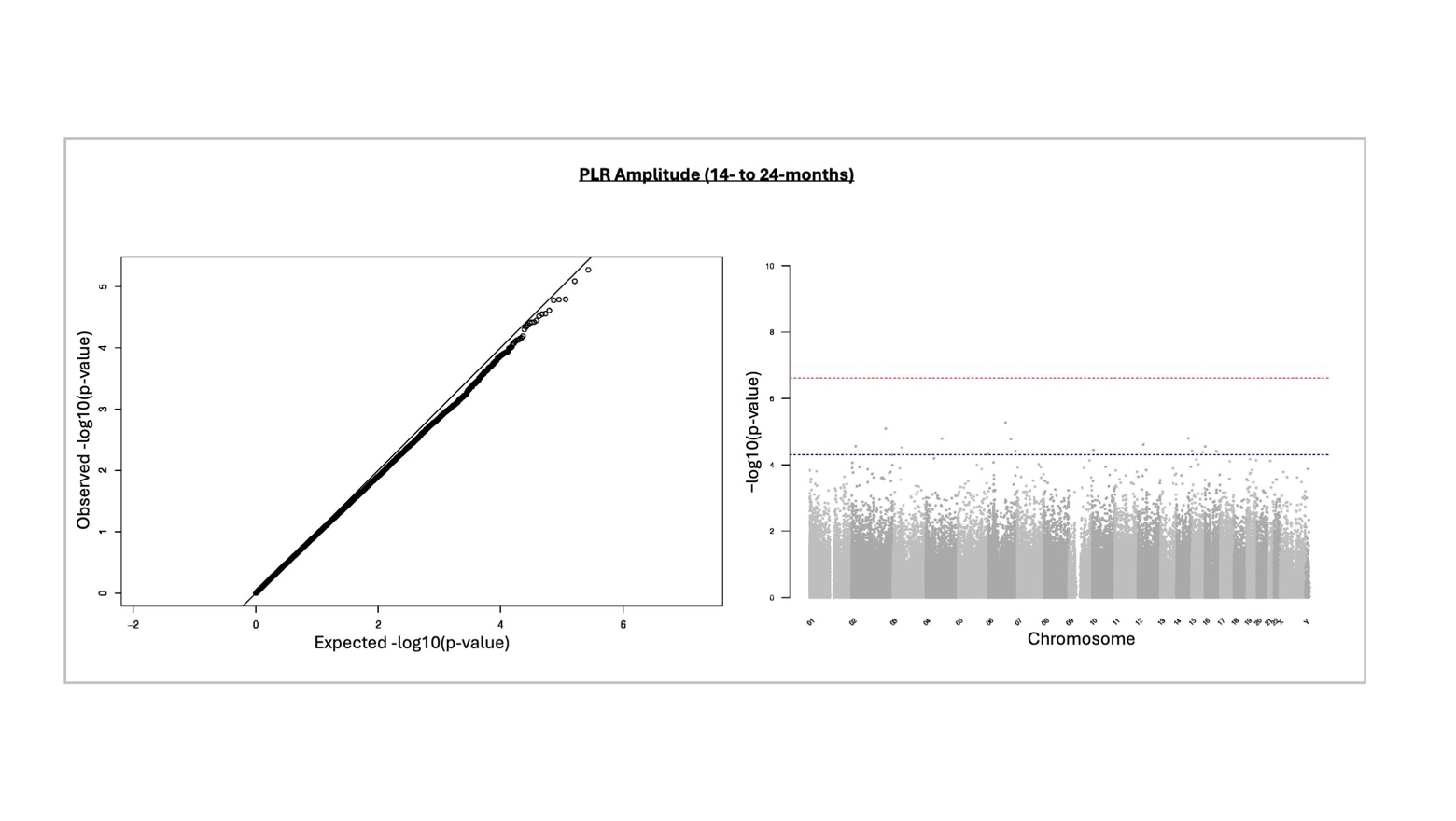
**

SM 4 Figure 10. Q-Q plot (left) and Manhattan plot (right) of results from 14- to 24-month PLR amplitude EWAS

## SM 4.2: EWAS probes at discovery p-value threshold (p < 5 × 10^-5^) or above.

See SM 4 Table 1 in the accompanying excel document for the full list of probes found to be significantly associated in each EWAS discovery p-value threshold *(p*< 5 × 10^-5^) or above.

## SM 4.3: Significantly associated DMR probes

See SM 4 Table 2 in accompanying excel document for full list of DMR found to be significantly associated.

# SM 5: Downstream exploratory analysis.

## SM 5.1: Gene Ontology input

See SM 5 Table 1 in accompanying excel document lists genes explored in Gene Ontology analysis for each phenotype.

## SM 5.2: Gene Ontology results

See SM 5 Table 2 in accompanying excel document lists significant terms found in Gene Ontology analysis for each phenotype.

## SM 5.3: SFARI gene comparison

See SM 5 Table 3 in accompanying excel document lists probes annotated to genes listed in the SFARI database (with corresponding SFARI gene-score) and associated phenotype.

# References

1 Ozonoff S, Young GS, Carter A, Messinger D, Yirmiya N, Zwaigenbaum L *et al.* Recurrence risk for autism spectrum disorders: A baby siblings research consortium study. *Pediatrics* 2011; **128**. doi:10.1542/peds.2010-2825.

2 Nyström P, Gliga T, Jobs EN, Gredebäck G, Charman T, Johnson MH *et al.* Enhanced pupillary light reflex in infancy is associated with autism diagnosis in toddlerhood. *Nat Commun* 2018; **9**. doi:10.1038/s41467-018-03985-4.

3 Fish LA, Nyström P, Gliga T, Gui A, Begum Ali J, Mason L *et al.* Development of the pupillary light reflex from 9 to 24 months: association with common autism spectrum disorder (ASD) genetic liability and 3-year ASD diagnosis. *J Child Psychol Psychiatry* 2021; **62**. doi:10.1111/jcpp.13518.

4 Nyström P, Falck-Ytter T, Gredebäck G. The TimeStudio Project: An open source scientific workflow system for the behavioral and brain sciences. *Behav Res Methods* 2016. doi:10.3758/s13428-015-0616-x.

5 R Core Team. R Development Core Team. *R: A Language and Environment for Statistical Computing* 2016; **55**.

6 RStudio Team. RStudio: Integrated Development for R. 2022.

7 Bergamin O, Bridget Zimmerman M, Kardon RH. Pupil light reflex in normal and diseased eyes: Diagnosis of visual dysfunction using waveform partitioning. *Ophthalmology* 2003; **110**. doi:10.1016/S0161-6420(02)01445-8.

8 Hellmer K, Nyström P. Infant acetylcholine, dopamine, and melatonin dysregulation: Neonatal biomarkers and causal factors for ASD and ADHD phenotypes. *Med Hypotheses* 2017; **100**. doi:10.1016/j.mehy.2017.01.015.

9 Hall CA, Chilcott RP. Eyeing up the future of the pupillary light reflex in neurodiagnostics. Diagnostics. 2018; **8**. doi:10.3390/diagnostics8010019.

10 Barfield RT, Almli LM, Kilaru V, Smith AK, Mercer KB, Duncan R *et al.* Accounting for population stratification in DNA methylation studies. *Genet Epidemiol* 2014; **38**. doi:10.1002/gepi.21789.

11 Auton A, Abecasis GR, Altshuler DM, Durbin RM, Bentley DR, Chakravarti A *et al.* A global reference for human genetic variation. Nature. 2015; **526**. doi:10.1038/nature15393.

12 Zheng SC, Breeze CE, Beck S, Teschendorff AE. Identification of differentially methylated cell types in epigenome-wide association studies. *Nat Methods* 2018; **15**. doi:10.1038/s41592-018-0213-x.

## 
